# Bidirectional Mid-Infrared Metamaterial with Ultrabroadband Polarization-Selective Absorption and Transmission

**DOI:** 10.3390/nano16211348

**Published:** 2026-10-24

**Authors:** Zheng Qin, Yongzhou Zhao, Yutong Zhou, Haihong Yu, Yijun Zhang

**Affiliations:** 1 School of Intelligence Science and Technology, Nanjing University of Science and Technology, Nanjing 210094, China; 2 Jiangsu Fasten Optoelectronics Technology Co., Ltd., Jiangyin 214400, China; 3 School of Optoelectronic Engineering, Chongqing University of Posts and Telecommunications, Chongqing 400065, China; 4 Physical Department, Changchun University of Science and Technology, Changchun 130012, China; 5 School of Electronic and Optical Engineering, Nanjing University of Science and Technology, Nanjing 210094, China

**Keywords:** metamaterial absorber, polarizer, bidirectional operation, ultrabroadband, high extinction ratio

## Abstract

Optical metamaterials provide a versatile platform for manipulating the amplitude, phase, and polarization of light at subwavelength scales and are therefore attractive for compact polarization-control devices. However, simultaneously realizing broadband polarization-selective absorption and transmission with bidirectional operation remains challenging. Here, we propose a bidirectional mid-infrared polarization-selective metamaterial comprising multilayer strip resonators integrated with a composite Au grating. For x-polarized incidence, the final bidirectional structure maintains an absorptance above 80% from 3.95 to 12.15 μm, with an average absorptance of 90.4%. For the orthogonal y-polarized incidence, the average transmittance reaches approximately 94% over the same spectral range. The device exhibits broadband polarization selectivity, with average absorption and transmission extinction ratios of 16.5 dB and 42.6 dB, respectively. Electromagnetic-field and structural analyses indicate that the broadband x-polarized absorption is associated with multiple localized resonances together with lattice- and Fabry–Pérot-cavity-related contributions, whereas the high y-polarized transmission is primarily enabled by the polarization-selective response of the Au grating. The symmetric configuration preserves these complementary polarization responses for illumination from either side, providing a route toward broadband multifunctional polarization control in the mid-infrared.

## 1. Introduction

Conventional infrared polarization devices based on birefringent crystals, wire-grid polarizers, or multilayer films are often limited by bulky configurations, material dispersion, operating bandwidth, and integration compatibility. Metamaterials and metasurfaces offer an alternative approach by enabling flexible control of the amplitude, phase, polarization, and propagation of light through engineered subwavelength structures [1,2,3]. Their compact form factor and structural design freedom make them attractive for multifunctional and integrated infrared polarization devices [4,5,6,7].

Among metamaterial-based polarization devices, subwavelength metallic gratings are particularly useful for controlling orthogonal polarization components because of their strong structural anisotropy [8]. Incorporating such gratings into metal–insulator–metal (MIM) configurations can produce polarization-selective absorption through polarization-dependent electromagnetic resonances [9,10,11], and their integration with infrared detectors further enhances polarization sensitivity. [12,13]. Similarly, anisotropic metamaterials can exploit distinct localized electromagnetic modes along different polarization directions to achieve high-contrast polarization-selective responses [14,15,16,17]. In addition, the polarization-dependent boundary response of metallic gratings can be exploited for selective transmission [18,19,20]. Furthermore, by tailoring the amplitude response and phase retardation of two orthogonal electric-field components, functions such as linear polarization rotation and linear-to-circular polarization conversion can be achieved [21,22,23,24]. Despite these advances, simultaneously combining broadband absorption for one linear polarization with high transmission for the orthogonal polarization in a single subwavelength platform remains challenging.

In addition to broadband and multifunctional polarization manipulation, bidirectional operation is also highly desirable for practical infrared optical systems. In practical applications, infrared optical components may be illuminated from either side depending on the optical path and system configuration. Therefore, maintaining consistent optical performance under opposite illumination directions can enhance device flexibility and simplify integration into complex optical systems. The optical responses of conventional asymmetric metal–dielectric metamaterials are usually strongly dependent on the illumination direction, making it difficult to maintain consistent polarization-selective performance when the device is flipped or operated under bidirectional light propagation. Although several studies have demonstrated metamaterial devices with bidirectional capabilities [25,26,27,28], simultaneously achieving high absorptance, high transmittance, a large polarization extinction ratio, and direction-independent operation over a broad spectral range remains challenging.

Here, we propose a broadband mid-infrared bidirectional polarization-selective metamaterial device consisting of a composite grating structure integrated with multilayer strip resonators. The device achieves efficient broadband absorption for x-polarized light while maintaining high transmission for the orthogonal y-polarized light and attains high polarization extinction ratios over a broad spectral range, representing a further improvement in overall performance compared with our previously reported structure [29,30]. Electromagnetic-field and structural analyses are used to identify the resonant contributions responsible for the broadband absorption and the grating-mediated mechanism responsible for the high transmission. We further examine the angular response and the influence of substrates with different refractive indices. Finally, a configuration symmetric along the propagation direction is introduced to realize the same polarization-selective functionality for illumination from either side, thereby expanding the application potential of the device in bidirectional IR polarization manipulation and integrated optical systems.

## 2. Materials and Methods

**Figure 1:**
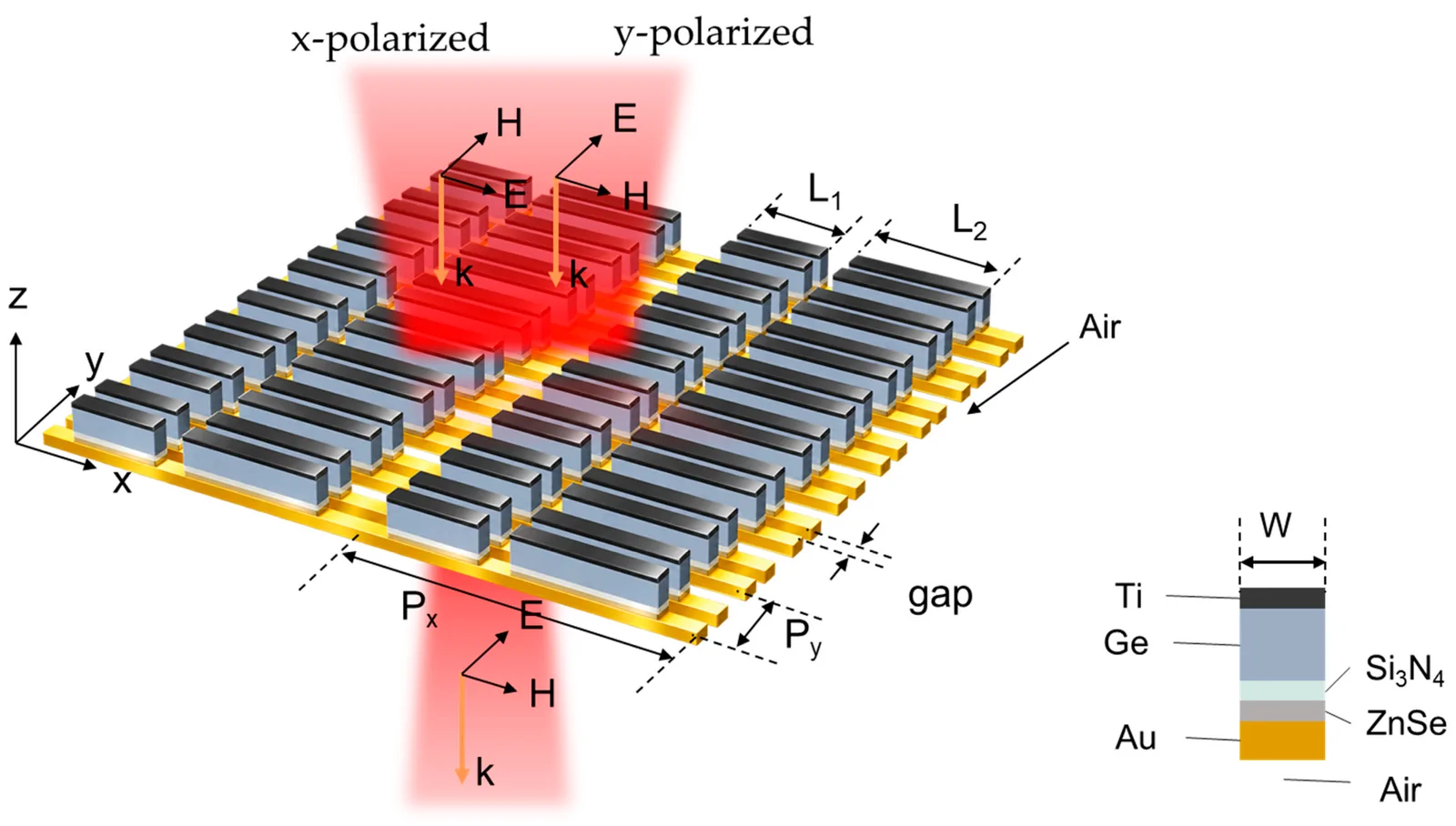
Schematic illustration of the proposed metamaterial device. Here, Px = 4 μm, Py = 1 μm, L_1_ = 1.1 μm, L_2_ = 1.75 μm, W = 200 nm, gap = 200 nm; the thicknesses of each layer are Au (200 nm), ZnSe (100 nm), Si_3_N_4_ (100 nm), Ge (400 nm), and Ti (100 nm).

As illustrated in Figure 1, the proposed metamaterial consists of a bottom gold grating extending along the x direction, with each unit cell along the y direction containing a pair of gratings. Each gold grating has a width of 200 nm, a thickness of 300 nm, and a gap of 200 nm from its neighbor. Stacked sequentially on top of the grating are ZnSe (100 nm), Si_3_N_4_ (100 nm), Ge (400 nm), and Ti (100 nm) layers, which together form a resonant cavity. The width of the resonator along the y direction matches that of the underlying Au grating, and two resonator units of different sizes are arranged alternately along the x direction, with *L*_1_ = 1.1 μm and *L*_2_ = 1.75 μm, respectively. The periods along the x and y directions are *P_x_* = 4 μm and *P_y_* = 1 μm, respectively.

These materials were selected because they are widely used in infrared optical devices, facilitating integration with existing optical platforms. Moreover, each material offers distinct advantages for achieving the desired optical functionalities. Specifically, Ti is commonly employed in broadband infrared metamaterial absorbers owing to its strong optical losses. Ge and ZnSe serve as high- and relatively low-refractive-index dielectric materials, respectively, enabling effective control of the electromagnetic response. Si_3_N_4_ provides additional dielectric absorption, while Au is widely used in the design of subwavelength metallic gratings.

The proposed metamaterial can be fabricated using conventional thin-film deposition and lithographic techniques. First, dielectric and metallic layers can be deposited onto an infrared-transparent substrate using sputtering, evaporation, or chemical vapor deposition, as appropriate. The patterned strip resonators and Au gratings can then be fabricated using electron-beam lithography, followed by metal deposition and lift-off.

The performance of the proposed metamaterial device was evaluated using Lumerical FDTD Solutions (2025). A linearly polarized plane wave is normally incident along the z direction, with the polarization defined such that the electric field is along the x direction for x-polarized light and along the y direction for y-polarized light. When a linearly polarized plane wave is normally incident, the boundary condition in the z direction is set to PML, while the boundary conditions in the x and y directions are set to periodic. The refractive indices of Au, ZnSe, Si_3_N_4_, Ge, and Ti were taken from refs. [31,32,33]. The reflectance R and transmittance T were directly computed by the software, and the absorptance was obtained as A = 1 − R − T.

## 3. Results and Discussion

**Figure 2:**
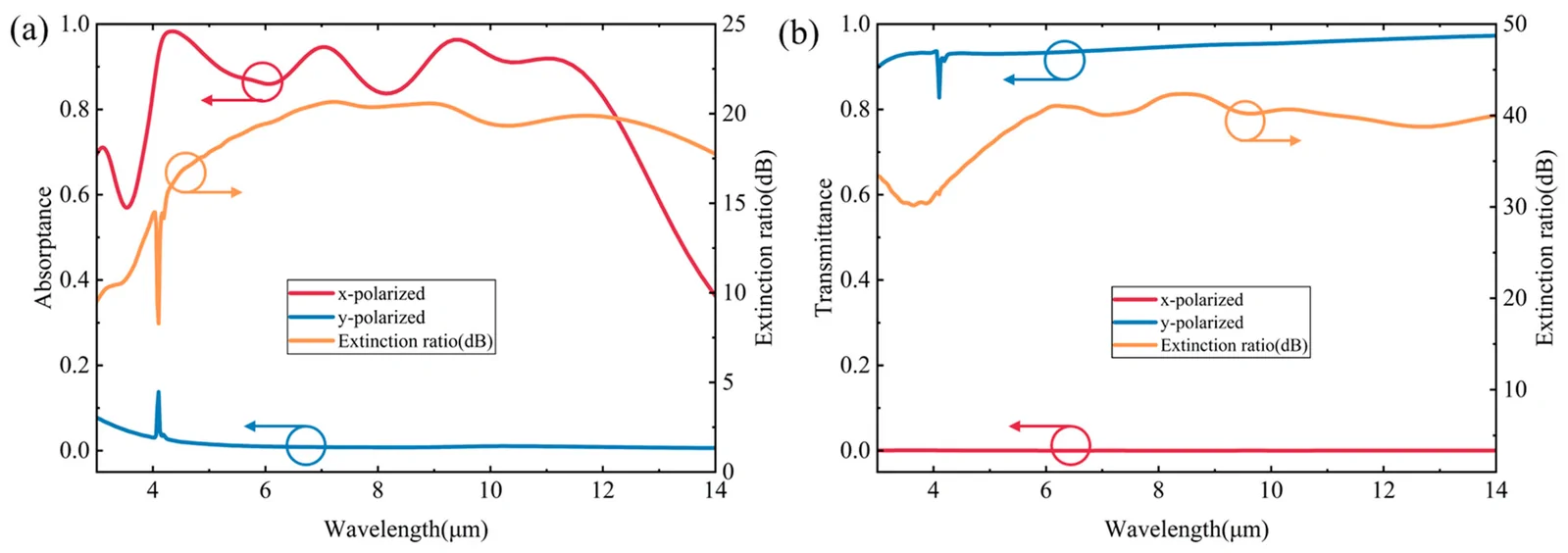
(**a**) Absorption and (**b**) transmission spectra of the proposed metamaterial under x- and y-polarized light incidence. Here, we use circles and arrows of the same color to indicate the y-coordinates of the spectra.

Figure 2 presents the spectral response of the metamaterial device under linearly polarized illumination. Under x-polarized light incidence, the device behaves as a broadband high absorber: the absorptance exceeds 80% over a broad spectral range of 3.95–12.15 μm, with absorption peaks at wavelengths of 4.3, 7, 9, and 11 μm, and the average absorptance within this band reaches 90%. The operating bandwidth is 8.2 μm, corresponding to a relative bandwidth of 102%, defined as $\frac{(\lambda_{1}-\lambda_{2})\times 2}{\lambda_{1}+\lambda_{2}}\times 100\%$.

The x-polarized transmittance remains below 0.05% across the operating band, indicating strong suppression of transmission by the grating–resonator structure. In contrast, for y-polarized incidence, the response closely follows that of the bare Au grating, with an average transmittance of approximately 94%, compared with approximately 97% for the grating alone. A narrow spectral feature near 4.1 μm is associated with a surface lattice resonance (SLR) [34] and produces an absorption feature of approximately 10%. Beyond 4.7 μm, the y-polarized absorptance remains below 2%, despite the intrinsic long-wavelength loss of Si_3_N_4_, consistent with the weak interaction of this polarization with the strip-like Si_3_N_4_ region reported in our previous work [29].

The polarization-selective performance was quantified by the absorption extinction ratio (AER $=10{log}_{10}(\frac{A_{x-polarized}}{A_{y-polarized}})$) and the transmission extinction ratio (TER $=10{log}_{10}(\frac{T_{y-polarized}}{T_{x-polarized}})$). Within the operating band, the average absorption extinction ratio (AAER $=\frac{\int_{\lambda_{1}}^{\lambda_{2}}AER(\lambda )d\lambda }{\lambda_{2}-\lambda_{1}}$) reaches approximately 19.6 dB. Furthermore, benefiting from the large transmittance contrast between y and x polarizations, the average transmission extinction ratio (ATER $=\frac{\int_{\lambda_{1}}^{\lambda_{2}}TER(\lambda )d\lambda }{\lambda_{2}-\lambda_{1}}$) is as high as 40 dB, polarization-filtering performance superior to that of previously reported wire-grid polarizers [19].

**Figure 3:**
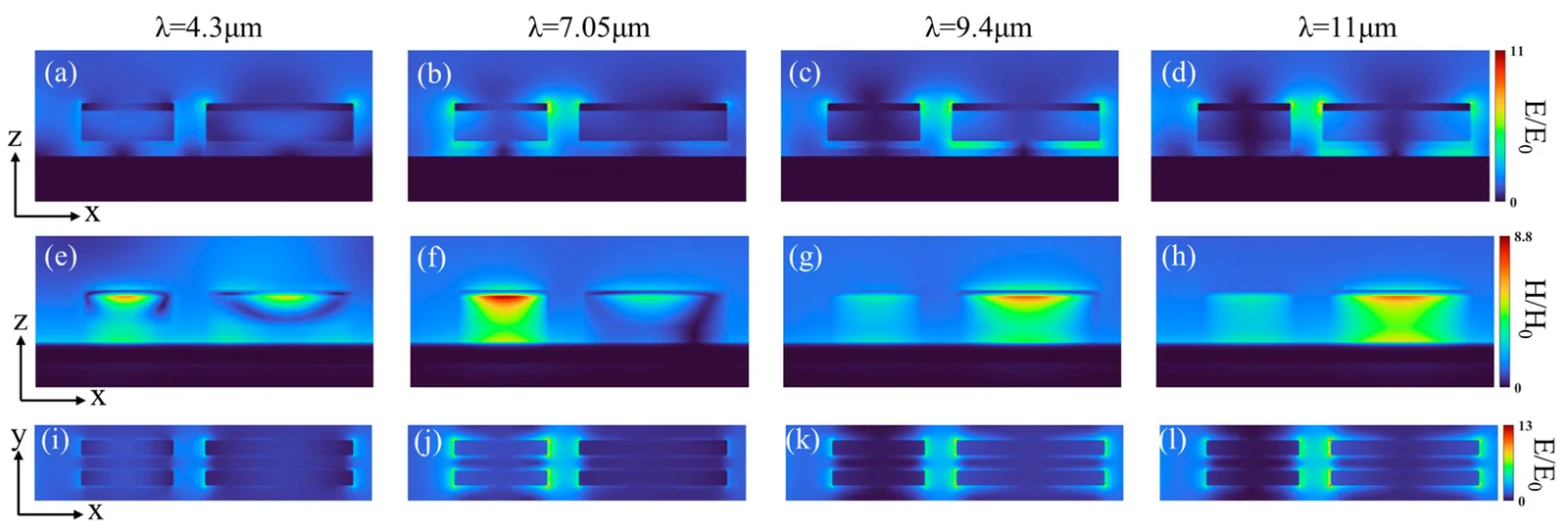
Distributions of the electric field (|E|) and magnetic field (|H|) at the absorption peak wavelengths (4.3, 7.05, 9.4, and 11 μm) under x-polarized light incidence. Electric field distribution in the xz-plane at a wavelength of (**a**) 4.3 μm, (**b**) 7.05 μm, (**c**) 9.4 μm, and (**d**) 11 μm; magnetic field distribution in the xz-plane at a wavelength of (**e**) 4.3 μm, (**f**) 7.05 μm, (**g**) 9.4 μm, and (**h**) 11 μm; electric field distribution in the xy-plane at a wavelength of (**i**) 4.3 μm, (**j**) 7.05 μm, (**k**) 9.4 μm, and (**l**) 11 μm.

To elucidate the physical mechanism underlying the broadband high absorption of x-polarized light, the electric- and magnetic-field distributions at four representative absorption peaks were calculated, as shown in Figure 3. At the absorption peaks at 7.05, 9.4, and 11 μm, pronounced local-field enhancement can be observed. The electric fields are mainly concentrated at the edges of the Ti resonators [Figure 3b–d,j–l], whereas the magnetic fields are predominantly confined within the dielectric layers beneath the resonators [Figure 3f–h]. Such strong electromagnetic-field localization indicates that these three absorption peaks are mainly associated with localized surface plasmon resonance (LSPR) [35] modes supported by the resonators.

Since the resonance wavelength of an LSPR mode is closely related to the lateral size of the resonator, smaller resonators generally support resonances at shorter wavelengths, whereas larger resonators correspond to longer-wavelength resonances. At approximately 7.05 μm, the enhanced electric field is mainly localized at the edges of the shorter resonator on the left, while the magnetic field is concentrated in the dielectric layer beneath it, indicating that this absorption peak is primarily associated with the LSPR mode supported by the shorter resonator. In contrast, at 9.4 and 11 μm, the enhanced electric fields are mainly distributed around the edges of the longer resonator. The highly similar electromagnetic-field distributions at these two wavelengths suggest that both long-wavelength absorption peaks are closely related to resonant modes supported by the longer resonator.

**Figure 4:**
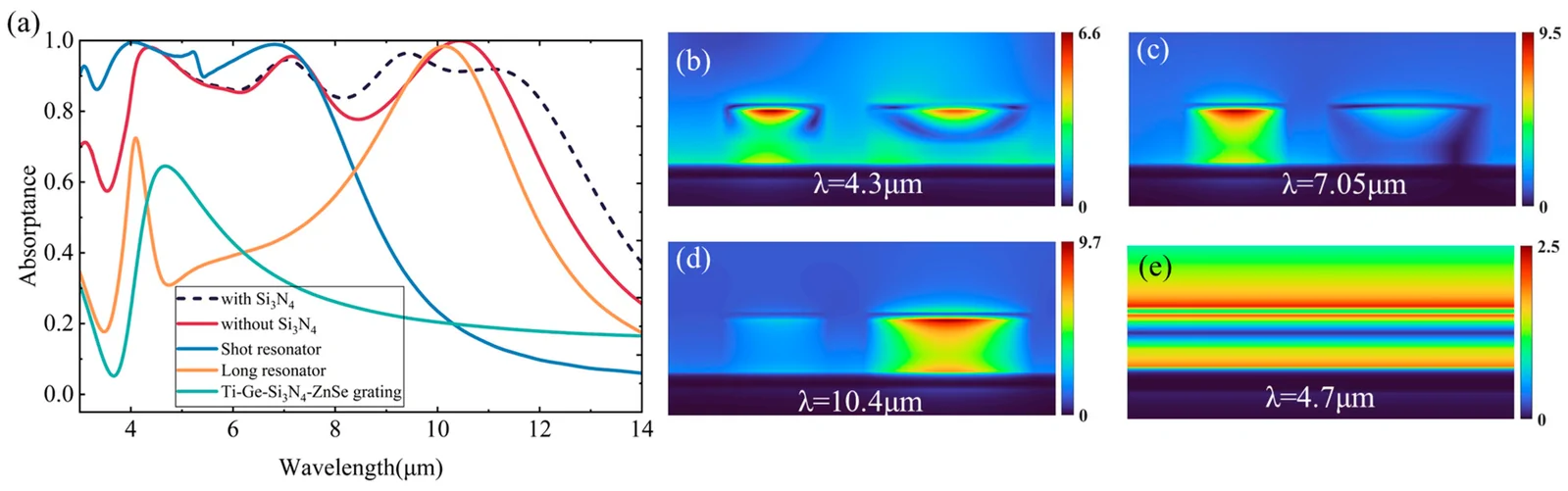
(**a**) x-polarized light absorptance spectra of metamaterials with different constituent structures. (**b**–**d**) Magnetic-field distributions at the three absorption peaks (4.3, 7.05, and 10.4 μm) of the structure without the Si_3_N_4_ layer. (**e**) Magnetic-field distributions at the absorption peak of the complete Ti–Ge–Si_3_N_4_–ZnSe–Au five-layer structure.

To further clarify the origin of the two closely related absorption peaks in the long-wavelength region, we compared the x-polarized light absorption spectra of several modified structures, as shown in Figure 4a. When the Si_3_N_4_ layer is removed and replaced by ZnSe while keeping the total thickness of the device unchanged, only three absorption peaks remain. The first two peaks nearly coincide with those of the original structure containing Si_3_N_4_, whereas the two original long-wavelength peaks merge into a single absorption peak located at approximately 10.4 μm. In addition, the overall absorptance in the long-wavelength region is slightly reduced after removing the Si_3_N_4_ layer. Figure 4b–d present the magnetic-field distributions at the three absorption peaks of the structure without the Si_3_N_4_ layer. These field patterns closely resemble the corresponding modes observed in the original structure shown in Figure 3, indicating that removal of the Si_3_N_4_ layer does not fundamentally alter the nature of the dominant resonant modes. Therefore, the enhanced long-wavelength absorption and the splitting of a single long-wavelength resonance into two absorption peaks in the original structure can be mainly attributed to the epsilon-near-zero (ENZ) response and relatively strong material loss of Si_3_N_4_ in this spectral region. The interaction between the Si_3_N_4_ layer and the localized mode supported by the longer resonator further modifies the long-wavelength resonance behavior and enhances electromagnetic energy dissipation.

Figure 4a further compares structures containing only the shorter or only the longer resonator. The peaks near 7.05 and 10.4 μm are predominantly associated with the shorter and longer resonators, respectively. By contrast, the feature near 4.3 μm is relatively insensitive to resonator length, suggesting a mechanism distinct from the localized modes discussed above. As shown in Figure 3a,e,i, both the electric and magnetic fields exhibit a certain degree of localization and enhancement around the edges of the two resonators and in the dielectric regions beneath them. However, the field enhancement is considerably weaker than that observed at the longer-wavelength absorption peaks, with the maximum intensity being less than half of that at the other resonances. This indicates that the absorption peak at 4.3 μm is not dominated by a strong localized surface plasmon resonance, but instead originates from the cooperative contribution of two distinct resonant modes. One contribution arises from the lattice resonance supported by the periodic array. This collective resonance results from diffractive coupling among neighboring unit cells, and its resonance wavelength is mainly determined by the periodicity along the x direction and is on a wavelength scale comparable to Px. Therefore, the absorption near 4.3 μm is relatively insensitive to variations in the dimensions of the individual resonators but is closely associated with the periodic arrangement of the overall structure. The second contribution originates from the Fabry–Pérot (F–P) cavity mode supported by the multilayer resonant structure. To further verify this mechanism, as shown in Figure 4e, a composite grating consisting of the Ti–Ge–Si_3_N_4_–ZnSe–Au five-layer structure and extending infinitely along the x direction was constructed. Its absorption spectrum exhibits a pronounced F–P cavity resonance centered at approximately 4.7 μm, confirming that the multilayer structure itself supports a strong cavity resonance in this spectral region. Taken together, these results indicate that the pronounced absorption peak at 4.3 μm mainly originates from the cooperative coupling between the lattice resonance of the periodic array and the F–P cavity mode of the multilayer resonator.

When light propagates from one medium into another, interfacial reflection can be effectively suppressed if the electromagnetic impedances on the two sides are identical or closely matched. The condition for optimal impedance matching is Z≈1+i0, which requires the real part to approach unity while the imaginary part remains negligible. We further extracted the effective impedance of the metamaterial under illumination with x-polarized and y-polarized light, where *S*_11_ and *S*_21_ denote the reflection and transmission coefficients, respectively [36].

(1) $$Z=\sqrt{\frac{{\left(1+S_{11}\right)}^{2}-S_{21}^{2}}{{\left(1-S_{11}\right)}^{2}-S_{21}^{2}}}$$

**Figure 5:**
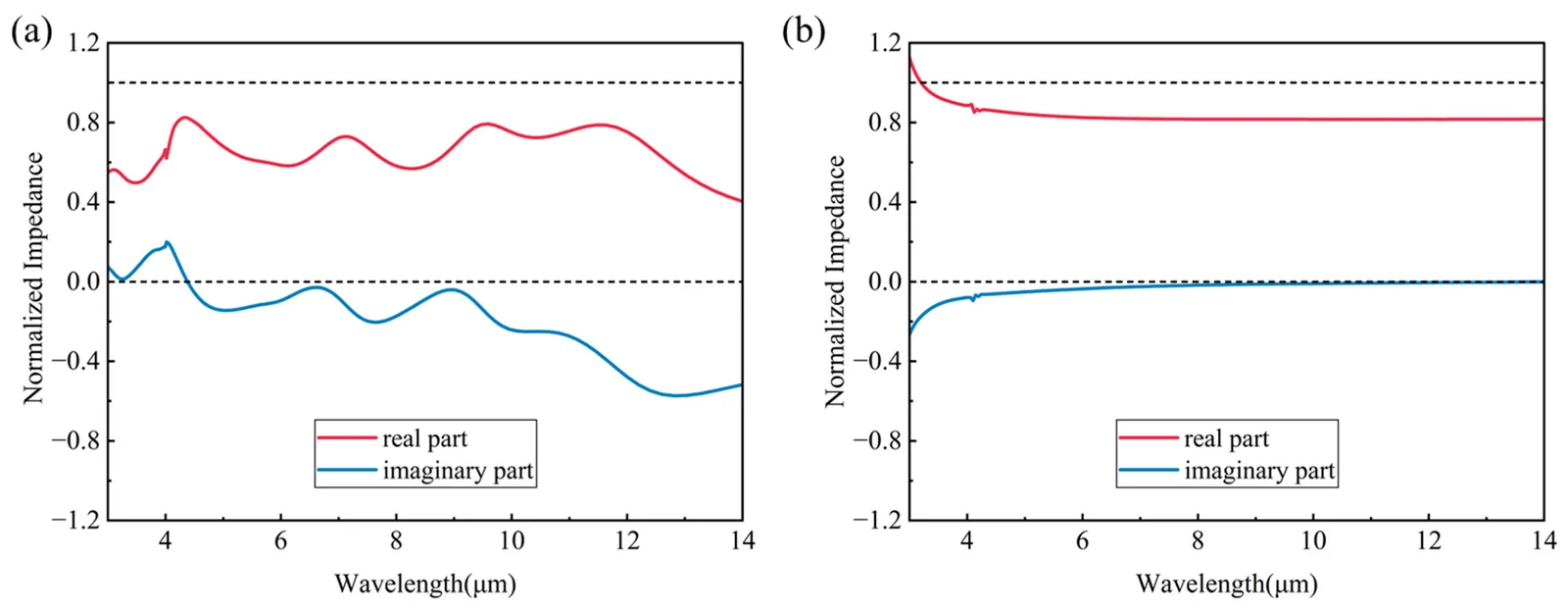
Normalized impedance of the metamaterial as functions of wavelength for (**a**) x-polarized light and (**b**) y-polarized light incidence. Here, the two dashed lines indicate impedance values of 1 and 0, respectively.

As shown in Figure 5a, the metamaterial is nearly impedance-matched to free space over a broad spectral range for x-polarized light incidence, effectively suppressing interfacial reflection. The strongly enhanced localized electromagnetic field subsequently induces pronounced Ohmic dissipation in the metallic regions and dielectric losses in the lossy dielectric, enabling the electromagnetic energy coupled into the structure to be efficiently absorbed. As shown in Figure 5b, for y-polarized light incidence, the metamaterial exhibits an even broader and better impedance match to free space, resulting in a reflectance lower than that of the x-polarized case. Because the y-polarized field does not efficiently excite the strongly dissipative resonant modes, its coupling to—and thus dissipation within—the device is weak, so that a high transmittance is retained.

**Figure 6:**
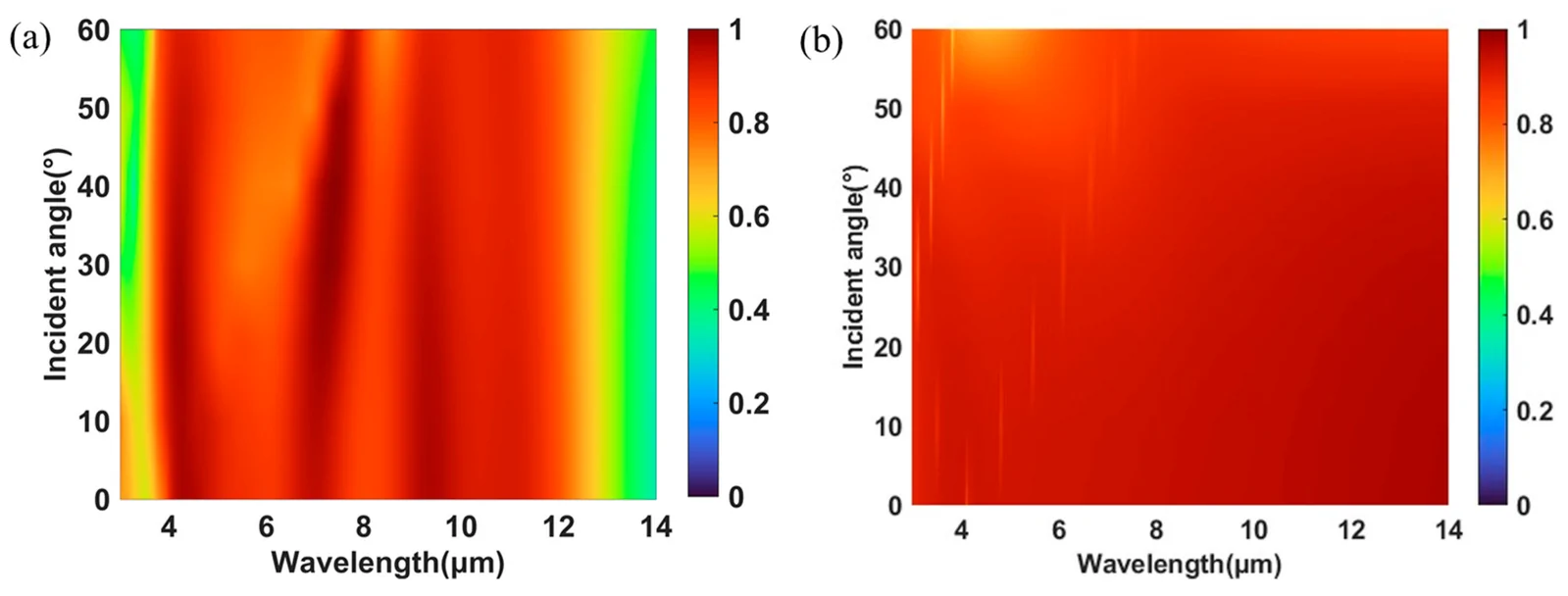
Angular dependence of (**a**) the absorptance for x-polarized light incidence and (**b**) the transmittance for y-polarized light incidence.

To further assess the robustness of the proposed metamaterial under varying illumination conditions, we investigated the angular dependence of its spectral response, as illustrated in Figure 6. Figure 6a shows the absorption spectra of x-polarized incident light at different incident angles. As the incident angle increases, the impedance matching between the metamaterial and free space gradually deteriorates, leading to a slight reduction in absorption efficiency. Nevertheless, the absorption spectra exhibit only minor variations, and the broadband high-absorption characteristics are largely maintained. Remarkably, even at an incident angle of 60°, the absorptance remains above 70% across the entire operating wavelength range, with an average absorptance exceeding 80%. These results demonstrate the excellent angular tolerance and robust broadband absorption performance of the proposed metamaterial under x-polarized illumination.

Figure 6b shows the spectral response of y-polarized light at different incident angles. As the incident angle increases, the surface lattice resonance undergoes splitting and exhibits pronounced angular dispersion, accompanied by a slight overall decrease in transmittance. Nevertheless, even at an incident angle of 60°, the average transmittance of y-polarized light remains approximately 83% within the operating wavelength range, indicating that efficient broadband transmission is well maintained. These results demonstrate that the proposed metamaterial preserves stable broadband x-polarized light absorption and high y-polarized light transmission over a wide range of incident angles, confirming its favorable angular tolerance and robust polarization-selective performance.

**Figure 7:**
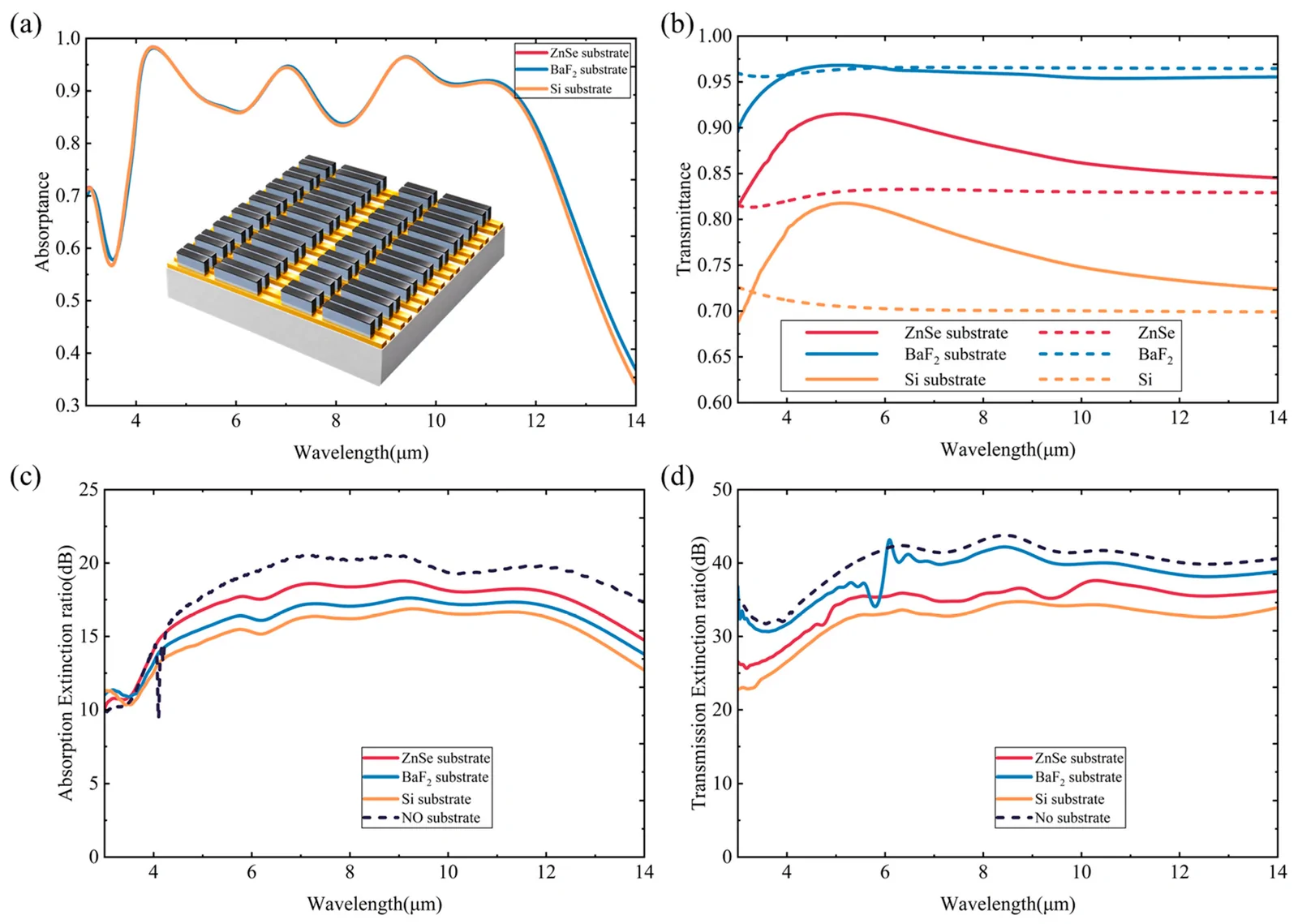
(**a**) Absorption spectra (inset shows a schematic diagram of a metamaterial structure with a substrate), (**b**) transmission spectra, (**c**) absorption extinction ratio, and (**d**) transmission extinction ratio of metamaterials with different substrate materials.

In conventional MIM absorbers, a continuous metallic back reflector largely isolates the resonant region from the substrate. In the present design, the continuous reflector is replaced by a patterned Au grating, allowing a fraction of the incident field to interact with the grating–substrate interface. Consequently, changes in substrate refractive index can modify the local dielectric environment, interfacial reflection, and transmission. To evaluate this effect, we simulated the structure on BaF_2_, ZnSe, and Si substrates (Figure 7).

As shown in Figure 7a, the absorption spectra of the device under x-polarized light illumination remain almost unchanged upon introducing different substrates, with the absorption curves for the three substrates nearly overlapping, indicating that variations in the substrate refractive index have little effect on the broadband x-polarized light absorption. This is mainly because the incident x-polarized light couples strongly with the upper multilayer resonant cavity and is largely absorbed before reaching the bottom of the structure; therefore, only a small fraction of the residual light arrives at the Au grating–substrate interface. For this unabsorbed portion of x-polarized light, its coupling into and transmission through the substrate is enhanced as the substrate refractive index increases.

The substrate has a stronger effect on the y-polarized response. As shown in Figure 7b, the transmittance of y-polarized light gradually decreases with increasing substrate refractive index because of the enhanced reflection at the substrate interface. To further distinguish the contribution of the metamaterial structure from that of the bare substrate interface, the transmittance spectra of the corresponding air–substrate interfaces without the metamaterial are also plotted as dashed curves. For the ZnSe and Si substrates, the transmittance of the bare air–substrate interface is lower than that of the metamaterial-integrated structure over most of the simulated wavelength range. This result indicates that the patterned structure also provides an antireflection contribution for y-polarized light.

The substrate also produces a modest increase in y-polarized absorption, which may arise from changes in the local dielectric environment and the interaction of reflected fields with the Au grating. Consequently, the absorption extinction ratio decreases as the substrate refractive index increases. As shown in Figure 7c, the AAER remains about 16 for the Si substrate. In addition, the substrate induces a blueshift of the y-polarized light-related lattice resonance, causing the original y-polarized light absorption peak and transmission dip to move outside the investigated spectral range.

The transmission extinction ratio is more sensitive to the substrate [Figure 7d]. Its average value decreases with increasing substrate refractive index and is approximately 33 for the Si substrate, substantially below the substrate-free value. Nevertheless, a large polarization contrast is retained for all three investigated substrates. The conclusion should therefore be limited to the substrates explicitly simulated rather than generalized to arbitrary substrate materials.

**Figure 8:**
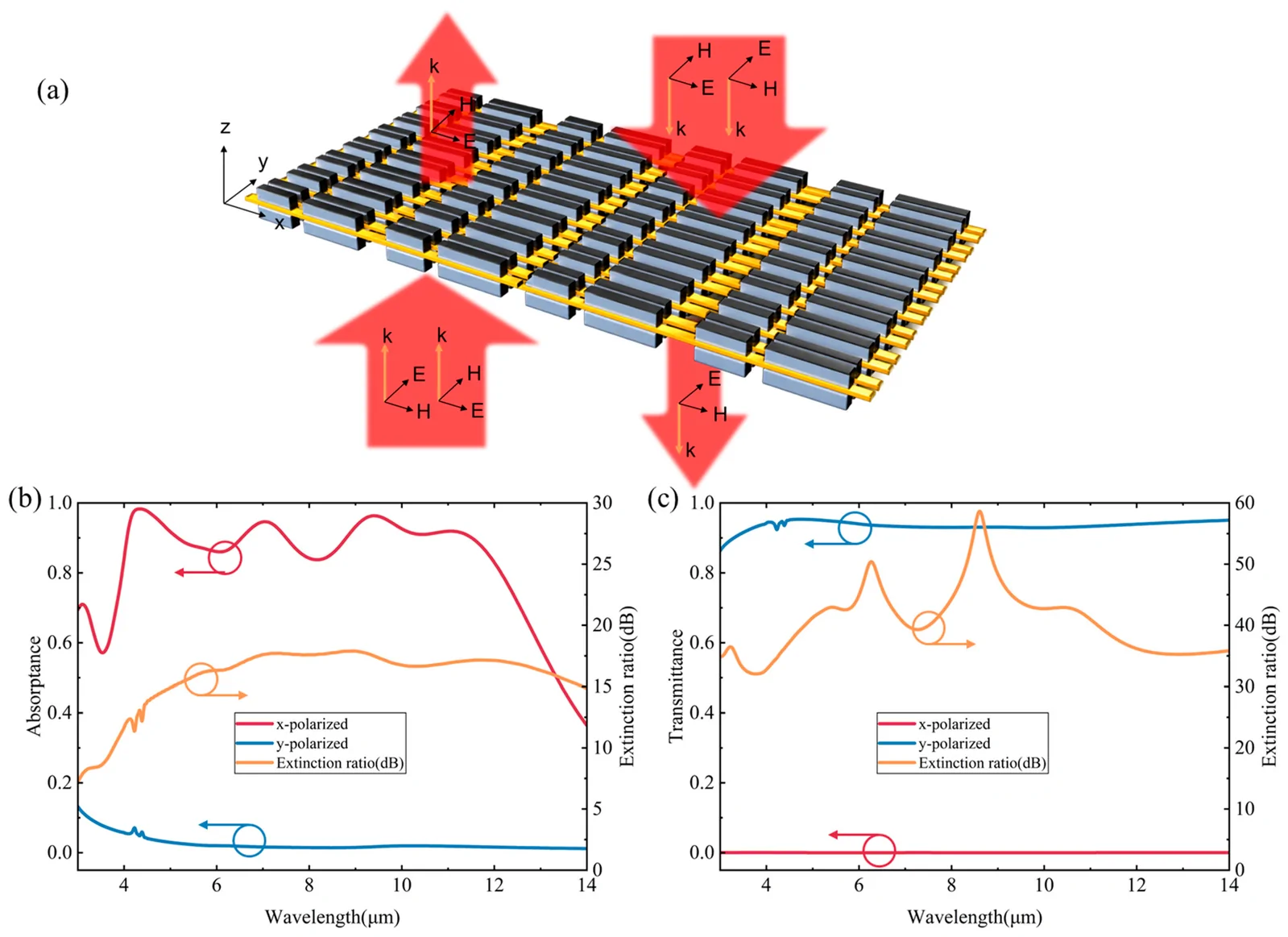
(**a**) Schematic illustration, (**b**) absorption spectra, and (**c**) transmission spectra of the bidirectional polarization-selective metamaterial.

The preceding results indicate that the polarization-selection mechanism can be retained when the structure below the Au grating is modified. On this basis, a propagation-direction-symmetric configuration was constructed by placing ZnSe–Si_3_N_4_–Ge–Ti resonators on both sides of the Au grating [Figure 8a]. This architecture is intended to provide the same x-polarized absorption and y-polarized transmission for illumination from either side.

As shown in Figure 8b, the absorption spectrum of the bidirectional polarization-selective metamaterial under x-polarized light illumination is nearly identical to that of the previously studied unidirectional structure. Over a broad spectral range of 3.95–12.15 μm, the absorptance for x-polarized light remains above 80%, with an average absorptance of 90.4%, demonstrating that the symmetric design does not noticeably compromise the original broadband absorption performance. On the other hand, owing to the larger overall thickness of the bidirectional structure, the light–matter interaction between the y-polarized light and the device is enhanced, leading to a slight increase in absorptance compared with the unidirectional structure. Accordingly, the absorption extinction ratio of the bidirectional structure is somewhat reduced; however, an average absorption extinction ratio of approximately 16.5 dB is maintained across the entire operating band, indicating that the device retains good absorption-based polarization selectivity. As shown in Figure 8c, the transmission characteristics of the bidirectional metamaterial under y-polarized light illumination are likewise essentially consistent with those of the unidirectional structure. The average transmittance for y-polarized light reaches approximately 94% over the 3.95–12.15 μm range, confirming that the symmetric architecture preserves the desirable transparent characteristics. The transmittance of x-polarized light is further suppressed after symmetrization due to the increased effective thickness of the composite grating, resulting in two pronounced transmission minima. Since the transmission extinction ratio is determined by the ratio between the transmittances of the two orthogonal polarization states, these two minima correspond to two distinct peaks in the transmission extinction-ratio spectrum. Over the entire operating wavelength range of 3.95–12.15 μm, the bidirectional polarization-selective metamaterial exhibits an average transmission extinction ratio of approximately 42.6 dB, while the minimum value remains as high as about 1800, demonstrating an exceptionally high and stable polarization contrast in transmission.

## 4. Conclusions

In summary, we have proposed a broadband bidirectional polarization-selective metamaterial based on a Ti–Ge–Si_3_N_4_–ZnSe–Au multilayer composite grating, which integrates strong absorption of x-polarized light and high transmission of y-polarized light within a single platform. The device operates over an exceptionally broad mid-infrared range of 3.95–12.15 μm, achieving average absorptance and transmittance exceeding 90% for the two orthogonal polarizations, together with pronounced polarization extinction. The average absorption and transmission extinction ratios reach 19.6 dB and 40 dB in the unidirectional configuration and remain as high as 16.5 dB and 42.6 dB, respectively, under bidirectional operation, demonstrating robust broadband polarization selectivity. The broadband absorption of x-polarized light arises from the synergistic excitation and coupling of multiple surface plasmon resonances and Fabry–Pérot cavity modes, whereas the high transmission of y-polarized light is primarily attributed to the polarization-filtering effect of the Au grating. The polarization-selective response also remains robust against variations in substrate refractive index, highlighting the structural tolerance of the proposed design. By combining broadband operation, bidirectional functionality, and strong polarization discrimination in a compact architecture, this work provides a versatile platform for manipulating mid-infrared light.

## Data Availability

The datasets used and/or analyzed during the current study are available from the corresponding authors on reasonable request.
